# Questioning the rhetoric of a ‘willing population’ in Finnish biobanking

**DOI:** 10.1186/s40504-019-0094-5

**Published:** 2019-05-27

**Authors:** Karoliina Snell, Heta Tarkkala

**Affiliations:** 1 Helsinki Collegium for Advanced Studies, University of Helsinki, Po Box 4, 00014 Helsinki, Finland; 2 Faculty of Social Sciences, University of Helsinki, Po Box 18, 00014 Helsinki, Finland

**Keywords:** Biobanks, Participation, Innovation policy, Finland, Informed consent, Public opinion

## Abstract

According to surveys and opinion polls, citizens in Nordic welfare societies have positive, supportive attitudes towards medical research and biobanking. In Finland, it was expected that this would result in the active biobank participation of patients and citizens. Indeed, public support has been rhetorically utilised as a unique societal factor and advantage in the promotion of Finnish biobanks, underlining the potential Finland offers for the international biomedical enterprise. In this paper, we critically analyse the use of notions such as ‘willing population’ and ‘engaged people’ in the promotion and legitimation of biobanking. First, there is a seeming contradiction between positive attitudes and actual participation rates, as biobanks have faced unexpected challenges in participant recruitment during the first years of their operations. As a result, the concept of a willing population was redirected to problematise the necessity of informed consent. Second, we question whether it is even meaningful to assume the existence of an informed and engaged population with regard to biobanking. Therefore, we suggest that it is problematic to talk about a willing population at the same time as the relevance of the informed consent system is being questioned by biobank actors and policy makers. We analyse this tension in relation to existing data on Finnish people’s attitudes, pointing out that positive, supportive views do not directly transform into high participation rates; nor do they justify the claims of policy makers and biobank proponents that people are willing to participate, when in fact surveys report that people know very little about biobanks.

## Introduction

Biobank activities in Finland are governed by the Biobank Act of 2013; since then ten biobanks have been established with high hopes that they will collect thousands of new prospective samples, ‘capturing all incomers’ (see Tupasela et al. 2015). Of these ten biobanks, nine are publicly funded: biobank of the National Institute of Health and Welfare, six clinical biobanks of hospital districts, a disease-specific biobank and the biobank of the Finnish Blood Service. The only private biobank, which is owned by a health care company, started its operations in 2018. In general, biobanks can be defined as social and technical arrangements for the collection, storage and distribution of tissue samples or other biological material and the medical and lifestyle data related to them. Due to advancements in genomics and in the technological capacity to work with larger data-sets, biobanks and transnational biobank networks are widely considered essential infrastructure for contemporary biomedical research, and vital elements of the knowledge-based bioeconomy (e.g., Yuille et al. 2007, Gottweis and Petersen 2008, Leonelli 2016). Currently, biobanks are not only part of the biomedical research structure but part of the landscape of new, large-scale, health data networks and ecosystems. Yet favourable public response and the willingness of individuals to donate samples and personal information are prerequisites for the collection and distribution of the bioinformation in biobanks (e.g. Levitt 2011, Levitt and Weldon 2005), and thus the enablers of economic development (Tarkkala et al. 2018, Hoeyer 2016).

Finland is known for high levels of trust in society (Gaskell et al. 2013, Väliverronen 2007), and Finns have also been reported to have higher levels of trust in public institutions and authorities, experts, scientific research and medical doctors than Europeans on average (Tupasela and Snell 2012, Eurobarometer 2010, Gaskell and Gottweis 2011, Snell et al. 2012). The opinions of Finns and the inhabitants of the other Nordic Welfare states – Sweden, Norway, Iceland and Denmark – are also among the most positive in Europe in regard to the possibilities of science and technology, medical research and biobanks (Eurobarometer 2010, 2014). These positive attitudes are often linked to the long history of the welfare state and its extensive public population and health registers, and comprehensive tissue collections, which have been utilized in research for decades (Tarkkala et al. 2018, Tupasela et al. 2015). Biobanks in Finland are for the most part associated institutionally and financially to the public sector and are framed as continuing this tradition of collecting samples and data. Therefore, references to ‘trust’, ‘willingness’ and ‘the positive attitudes of the population’, when it comes to research participation, can be considered an established narrative (Prainsack 2007) in Finland that contributes to the legitimation of science, medicine, technology and, concomitantly, biobanks. As Prainsack (2007) has further observed, when the rhetoric employed by biobank promotors corresponds with established narratives, this increases the likelihood of obtaining public support and trust.

Public support has been mentioned in health sector innovation strategies and visions as an important competitive advantage for Finland; the population has often been described as ‘willing’ to participate, which is seen as a critical factor in the potential success of Finnish biobanks (see, e.g., Ministry of Social Affairs and Health 2015, 20, Ministry of Social Affairs and Health 2016). Furthermore, labels of ‘willing’ or ‘engaged’ prevent associations with exploitation (e.g., Vora 2015). Consequently, this kind of reasoning – the significance of a willing and educated population in creating a success story of biomedical research – was used in Iceland about 20 years ago when Decode Genetics and IHDB were founded (see, e.g., Rose 2003, Tarkkala 2019). Yet many countries are also branded as ‘the place to be’ for biomedical or clinical studies based on a population that is presented as having an exceptionally useful genetic or social background in terms of uniqueness or diversity (see Tupasela 2017, Benjamin 2009, Ong 2016, Tarkkala and Tupasela 2018). Finland likewise exemplifies a country rhetorically framed as offering a competitive population for international biomedical research, in Finland’s case due to its biological homogeneity, but in this study we focus on a *social* characteristic that is deployed to advantage: the ‘willingness’ and supportive attitudes of its population (see also Rose 2003). Similar taken-for-granted assumptions about populations, right ways to organise biobanking or participation, and their implications can be observed in other national and multinational initiatives and in science and technology policy (Busby 2006, Woolley et al. 2016, Welsh and Wynne 2013).

When the first biobanks were founded in Finland in 2013–2015, it was expected that the positive attitudes of the country’s citizens towards research would result in their active participation. This did not materialise, however, and many biobanks struggled to attract donors. The recruitment process, and the informed consent system supporting it, were therefore identified as impeding the success of Finnish biobanks (Ministry of Social Affairs and Health 2016). Nevertheless, this recognition did not result in the abandonment or even problematisation of the rhetoric of a willing population. Instead, as we argue in this article, alongside its continued deployment in highlighting Finland’s competitiveness, the notion of a ‘willing population’ was re-harnessed to support the abandonment of informed consent. The argument goes that, as the majority of Finns are positive about biobanks, there should be no need to obtain formal consent (Ministry of Social Affairs and Health 2016); the required accumulation of samples and data could be secured by referring to the public good as a legislative base for collection. This turn towards abandonment of informed consent was a move to amend biobank legislation, which started almost immediately after the first law came into force and provided a facilitating environment. Further reasoning supporting the initiative related to the history of Finland as a Nordic welfare state and the long tradition of gathering register data. Biobank samples and data were presented as similar to, and therefore a continuation of, this tradition, which had not required consent in the past, making biobanking just business as usual.

We start from the contradiction between expectations of population enrolment in biobanks and actual participation rates. Moving on from there, we question the utilisation of notions such as ‘willing population’ and ‘engaged people’ to legitimise biobanking and biomedical research, claiming that such labels are neither empirically nor ethically defensible given that a large proportion of Finns do not know what a biobank is (Snell 2017). The public support for science and trust in medical experts identified in surveys do not equate with informed willingness to participate in biobanks nor knowledge on biobanks. We are not discussing the motivations of Finns to participate or not, but point out that it is questionable to talk about willingness if, rather than being specifically asked for their consent, people’s information and samples are made available to biobanking by default – by using legislation as the basis for data and sample collection. The article is motivated by concern about statements presented by biobank proponents and innovation policy makers, and how the statements are employed in strategies and argumentative communication related to promoting biobanking.

## Data and analysis

Our argument is based on analysis of three types of data: 1) Finnish reports and strategies related to biobanking from 2014 to 2016 (*n* = 5); 2) presentations and slideshows promoting Finnish biobanking from 2013 to 2017 (*n* = 22); and 3) material collected from the internet (including blogs, news and webpages) about biobanking from 2013 to 2018 (*n* = 15). The material has been gathered since 2013 for use in a range of research projects in which the authors have been involved, which have dealt with biobanks and biomedical infrastructures and policies (e.g., Snell et al. 2012, Tarkkala et al. 2018, Tarkkala 2019.). In these projects, we had noticed that the theme of a positive, trusting and willing population was a prominent narrative in public discussion about biomedicine, the biomedical R&D environment, science and biobanks. Wanting to examine it more thoroughly through the case of biobanking, we scanned a larger corpus of collected material and picked the data set described above for closer analysis on the basis that the texts used the narrative in question or referred to the Finnish population and its role in biobanking in some way. We identified and classified different manners of referring to the population and the terms and rhetorical tools that were used in connection with it – such as willing population, the positive attitudes of patients, trusting population, and tech-savvy Finns – and identified the contexts in which they were deployed. The narrative of the willing population in particular offers insights into what it promotes and legitimises. Thus we contribute to the discussions on policy and strategy rhetoric around biomedicine and its social end ethical implications (Corrigan and Tutton 2006, Woolley et al. 2016, Tupasela 2011).

In addition, analysis reflects existing research on knowledge and opinions towards biobanking in Finland (Sihvo et al. 2007, Tupasela and Snell 2012, Snell 2017, Gaskell et al. 2013, Snell et al. 2012), as well as statistics of participation rates obtained by personally requesting them from biobanks. Thus we do not analyse the motivations to participate as such. Analysis is restricted to Finland, but the article raises important points which also concern data-gathering and biobank initiatives elsewhere in terms of their socially sustainable development. Unpacking the premises and uses of rhetorical tools can make visible taken-for granted assumptions that guide and legitimise policies and their implementation.

## Positive, willing and engaged population

The rhetoric of a positive population has been utilized in public discussions of science and technology, and had an especially important role at the strategic level and in innovation policy documents in Finland throughout the 2000s (Snell 2009). In texts related to biomedicine, biobanks and genomics, a ‘positive attitude’ is closely correlated with willingness to participate in medical research and data repositories. In the Finnish Genome Strategy (Ministry of Social Affairs and Health 2015), written to promote and advance the usage and utilization of genomic data, it is said:

From a global perspective, Finland’s strengths include a high standard of healthcare, uniform treatment practices, reliable healthcare registers, a long tradition of high-quality genetic research, and the willingness of the population to participate in scientific research. (Ministry of Social Affairs and Health 2015, 12.)

Similarly, the Health Sector Growth Strategy (The Ministry of Employment and the Economy 2014, 33) states that “Finland also has patients who take a positive view of studies, which significantly promotes clinical research activities.” And in a report evaluating the integration of Finnish biobanks it is claimed that:

The potential and value proposition of biobanking efforts in Finland are unique due a number of key attributes, including (i) the Finnish population’s exceptional genetic founder characteristics, (ii) the depth, breadth, and decades-long track record of Finnish Electronic Health Records (EHR) linked to a unique personal ID number, (iii) the enactment of a progressive biobanking law, and (iv) the generally positive attitude vis-à-vis participation in biomedical research of a highly educated populus. (Ministry of Social Affairs and Health 2016, 5)

This kind of rhetoric has been used in Finland for decades (see Snell 2009, Tupasela 2007, Tarkkala 2019.) to suggest, for example, that Finns have played a key role in studies on monogenetic diseases (see, e.g., Peltonen et al. 1999, 1920). Besides the aforementioned strategy and policy documents, the positive and therefore willing population also makes an appearance in material promoting Finnish biobanking and the potential for biomedical research in Finland. One biobank touts Finland as an excellent location for biobanking in its webpages in the following terms:

Compared to many other countries, Finland's prerequisites for creating valuable biobanks are considered excellent. Finnish biobanks are supported by a uniform public health system, precise registration of medical history, a population register and citizens who have a positive attitude towards research work. (Auria Biobank 2013)

Sitra – an influential and independent fund reporting to Finnish parliament that is commissioned with the task of probing the future and promoting qualitative and quantitative economic growth – has prepared a slideshow entitled “Finland - Your testbed for research and medical innovation” (Sitra 2015), which has been employed by many prominent actors in the field of biobanking and biomedical research: biobank managers and professors as well as representatives from ministries and companies. Due to their broad dissemination, the slides can be considered highly influential promotional tools for Finland in the biomedical research market, both at national and international events. One of the five reasons listed in the slide sequence for why Finland is the most advanced testbed in the world, is its ‘engaged population’, a small twist on the willing population narrative. An engaged population, according to the Sitra slides, is constituted by four elements: high levels of education, a marked willingness to participate, trust in the authorities and tech-savvy people. This raises the question of how these elements foster engagement? Is trust in the authorities, for example, the primary quality of an engaged population?

The positive and willing population also features in the Finland Health webpages to promote the Finnish R&D environment in biomedicine and health technology. In one article on its site, the Finnish consent rates to having samples stored are regarded as ‘astoundingly high’:

Finland boasts an astoundingly high consent rate when it comes to their rate of consent: over 90% of Finns consent to donating their samples to a biobank upon request! ... Finns rank at the top of the charts when it comes to trusting institutions and valuing science. They understand the importance of science, and want to be involved in furthering research. The positive Finnish attitude towards research is only one of the reasons why Finland is the country for sample collecting. (FinlandHealth 2016)

Thus, high consent rates are accepted as a sign of positive attitudes; however, it is not apparent what type of consent the site is talking about, because, while 90% may agree ‘upon request’, how many patients actually receive such a request? A similar ambiguity is present in the abovementioned testbed slides that state that “95% of consent donators have given consent of their samples [sic]” without specifying what samples, what donors, and what consenting is in question. Firstly, it is difficult to construct a common consent rate for biobanks, each of which has a different method for gaining consent and represents a range of general clinical, disease-specific and research-based biobanks, while all are in different stages of operation: some have been collecting new samples for years while others have more or less just started. Secondly, almost immediately after the new biobanks began to collect new samples, it was noticed that receiving informed consent from patients to do so was anything but simple and easy. The first clinical biobank, for example, struggled to get a 25% return rate of informed consent forms.

## Concern over consent rates – creating another use for the willing population

The legislative arrangements during the first years of establishing biobanks form one background for the connection between the rhetoric of success and high consent rates. The Biobank Act provides Finnish biobanks with two routes to acquiring biological samples: integrating existing collections or collecting new prospective samples. These procurement methods entail different practices in terms of consent. Old research, clinical or diagnostic collections could be transferred to a new biobank within a certain timeframe merely by following a notification procedure – an advertisement in a newspaper has been considered sufficient (Soini 2016). The sample ‘donor’ can then opt out of the biobank if so desired. In contrast, new samples require informed and written consent. While there have been hardly any opt-outs from the old collections, nowadays referred as ‘legacy samples’, this cannot be regarded as *willingness* to participate. It is likely that many past donors have not seen the ads in the newspapers, and do not know that their old clinical and diagnostic samples have been transferred to a biobank; nor, possibly, do they know about their right to opt out. Furthermore, as mentioned above, most Finns do not know what biobanks are. Recent survey results show that only 40% of Finns have heard of them and, of those, 63% are considered to have insufficient understanding of them to make an informed decision (Snell 2017). Being a willing participant in a biobank requires, first of all, knowledge about their existence and, secondly, awareness of the possibility that one’s samples and related data can be stored in one. Incidentally, to put the legacy samples in context, it was estimated that over two million Finns would become participants in biobanks due to the transfer of old samples (Sosiaali- ja terveysministeriö 2007, 20), and millions of samples have since been transferred. Meanwhile, by the end of 2017, only about 126,000 informed consents had been acquired for prospective sampling (Biopankki.fi 2017).

Currently, the findings in surveys or qualitative studies of favourable attitudes towards medical research do not translate in a straightforward manner into high consent rates or participation in biobanks. Even though research shows consistently positive attitudes towards science and medicine in Finland, people either remain unaware of what biobanking is or they hold preconceptions and concerns about biobanking and medical research that require attention (Tupasela and Snell 2012). While research shows that educated citizens in particular have positive attitudes, it is also they who understand that there is a wider context of biobank research that needs to be addressed (Snell 2017). This is reflected in their concerns about the consequences of biobanking in society, and the issues of control, equality, transparency and commercialisation related to the storage and circulation of biobank information; these appear to be their main worries along with a general unease about the unknown future of biobanking (Snell et al. 2012, Tupasela and Snell 2012, Critchley et al. 2015, Meskus 2018).

As stated, gathering new, prospective samples or integrating new information and old samples requires informed consent from the donors. The consent rate has been disappointing for many hospital biobanks (Heino 2016). The early enthusiasm and rhetoric about a willing population that participates in biobanks rather swiftly attracted a simultaneous concern with consent rates; consequently, the consent process was framed as the problem hindering and slowing down the actualization of success in the field. Mentioned above, this was clearly stated in a report discussing the integration of Finnish biobanks:

An important current bottleneck is sample collection, which is hampered by the current informed consenting processes that have turned out to be less than optimally effective. (Ministry of Social Affairs and Health 2016, 9).

According to our biobank informants, only 20%–50% of the consent forms for new samples sent by mail to potential donors had been returned to the biobanks, although only a very small minority of the returned forms had been refusals. Thus people seemed unprepared to take a stand. Quantitative findings support this as one survey found 44% of respondents were interested in giving their samples to a biobank while 45% were uncertain (Snell 2017). Some biobanks have invested in ‘biobank ambassadors’, who tour hospital clinics to collect informed consent documents in person. This method has proved more successful numerically and Helsinki Biobank, for example, has reached its targets. This type of recruitment is, however, regarded as time and money consuming and therefore inefficient.

The National Institute of Health and Wellbeing (THL) has had a higher success rate in acquiring informed consent, as it recruits people to take part in health research projects and to the THL biobank. But their consent rate is based on people who have already decided to participate in a research project, after which it might be easier to accept biobank participation. Meanwhile, there has also been considerable discussion and concern about declining participation rates in epidemiological and clinical research projects (Mindell et al., 2015).

The report on the integration of biobanks that identified the bottleneck continues by providing another rhetorical use of the willing population, observing that if people are in general positive, seeking informed consent might not be the way forward. The report refers to planned legislation regarding the secondary use of health data.

Since a great majority of Finns is willing to provide biobank consent and samples, we need new ideas and resources to address this critical bottleneck. The planned change in legislation regarding a liberalization of the secondary use of data is one important step in the right direction in this regard. (Ministry of Social Affairs and Health 2016, 9)

The idea of the legislation is that all health data should be considered as register information gathered on legislative grounds and therefore an opt-out mechanism is preferable to informed consent. In a blog text from 2016 a prominent Finnish professor drew attention to the educated sector of the population and patients who want to participate in biobanks but have difficulties because of the highly bureaucratic consent process, suggesting that this is hindering biobank development in Finland. He also suggests the opt-out procedure as an attractive alternative to informed consent:

The solution most worthy of support would be, that by default, when a patient enters public health care, along with taking another blood sample a biobank sample would be taken, unless he specifically declines it. (Palotie, 2016)

Thus, despite the consent ‘bottleneck’, the positive population is still seen as one of the key strengths of prospective biobanking in Finland, with its ‘willingness’ serving as evidence that informed consent is actually not needed. Even the report identifying the bottleneck mentions the positive population as a key attribute of value creation for biobanks (Ministry of Social Affairs and Health 2016, 5). It is thus being proposed that the trope of the willing population be used to enable the dismantling of informed consent procedures based on the population’s extrapolated readiness to participate; this is regarded as the solution to the acknowledged difficulty in recruiting participants.

Johnsson et al. (2010, 1261) have suggested that the “willingness to participate in research” expressed in surveys “may not always predict actual participation rates” and thus, “the value of surveys in assessing factual willingness may thus be limited”. They add that this is why actual participation rates are higher than surveys indicate, because of factors present in face-to-face situations. Our analysis does not fully support these findings, however, as the actual consent rates were lower than expected. Van Zon et al. (2016) also mention lower actual participation rates in many biobank studies, arguing that people might be more prone to give a socially desirable answer to a hypothetical study costing the hypothetical participants no time or effort. We agree with Van Zon et al.’s assessment that hypothetical situations do not reflect real situations where people have to make concrete and possibly long-lasting decisions based on inadequate knowledge. Yet we also agree with Johnsson et al. that personal encounters increase participation rates, as has become evident in the recruiting procedures of some biobanks and clinics. Biobank ambassadors have been successful in face-to-face recruiting and consent forms acquired in disease-specific biobanks where the consent process is more personal have been high. One biobank manager describes the success of face-to-face recruiting as follows:

Consent is currently 98%. Most people from whom consent has been asked, have given it. Biobank operations have been received very positively… People want to be part of developing Finnish medicine. (HUS 2016)

The number of people formally consenting is indeed on the rise, from 3000 in 2014 to over 120,000 at the end of 2017 (Biopankki.fi 2017). The instigation of new biobanks as well as some large research projects that include biobank consent have contributed to these figures.

## Discussion

We do not question the positive attitudes towards science and medicine expressed by Finns and demonstrated in numerous surveys and research articles. We also acknowledge that the rising number of informed consents is an indicator of increasing familiarity with biobanking, as well as growing efforts and resources allocated to the recruitment of biobank participants. Thus, our aim is not to claim that people will not participate in biobanking in the long run. Instead, we have directed our critical gaze at the rhetoric that uses the idea of a positive and trusting population in a manner that we do not consider socially or ethically robust. Critical social analysis has to go beyond these kinds of established narratives and taken-for-granted assumptions that continue to circulate around biomedicine.

We approached out case from two viewpoints. First, we underlined how research on public opinion has consistently demonstrated that biobanking has been, and still is, an alien concept for Finns (Sihvo et al. 2007, Snell 2017). Despite this, the rhetoric of a willing and engaged population continues to be deployed; indeed, it even appeared in the early phases of Finnish biobanks when there was no evidence either way about consent rates. Second, we were concerned by how the argumentation of a willing and engaged population is being used as evidence to circumvent the consent process in favour of an opt-out model.

Supporting and positive attitudes or high levels of general trust in a given society are not in themselves straightforward indications of people’s actual willingness and preparedness to participate in biobanking. During the first years of biobank sample collecting in Finland, it was acknowledged that attaining informed consent from patients and citizens was creating a bottleneck and thus the consent procedure came to be regarded as hindering the development of biobanking and biomedicine. Even though the low participation rate was seen as a challenge, the actors and proponents of biobanking framed it as a practical issue, suggesting that the consent process was of the wrong kind. The actual support and willingness of people to participate was not put under scrutiny.

Simultaneously, the informed consent previously seen as a prerequisite for responsible and transparent biobanking practices seemed to lose its significance. We suggest that it is not valid to portray the consent process as unnecessary simply because people do not act as was expected and hoped of them (see also Sterckx et al. 2016). It might be meaningful to consider whether this situation and reasoning is indicative of what happens as innovation policy increasingly becomes a key framing of personalized medicine initiatives (see Tarkkala et al. 2018). As the willing population continues to figure in different strategy materials related to biobanking, personalised medicine and health sector innovations, it would be wise to investigate the grounds on which a population may be regarded as ‘willing’ in the first place. We acknowledge that an opt-out system has advantages - it is a more efficient way to collect samples and data and can also be preferred by biobank participants. We have not aimed to take a stance for or against informed consent or opt-out as such, but want to highlight how certain rhetoric is embraced to drive certain and sometimes changing ends. In our case, the rhetoric of willing population is utilised not to convince public to support biobanks but to convince of the existing support for biobanks and their success.
